# Patient Sharing of Digital Health Data in the Veterans Health Administration: Cross-Sectional Analysis

**DOI:** 10.2196/80517

**Published:** 2026-03-26

**Authors:** Mark S Zocchi, Jessica M Lipschitz, Felicia R Bixler, Stephanie A Robinson, Bella Etingen, Timothy P Hogan, Ndindam Ndiwane, Saige Calkins, Terry Newton, Nilesh Shah, Ben Kragen, Alexander S Young, Stephanie L Shimada

**Affiliations:** 1 eHealth Partnered Evaluation Initiative VA Bedford Healthcare System Bedford, MA United States; 2 Center for Health Optimization and Implementation Research VA Bedford Healthcare System Bedford, MA United States; 3 Division of Health Informatics and Implementation Science Department of Population and Quantitative Health Sciences UMass Chan Medical School Worcester, MA United States; 4 Department of Psychiatry Brigham and Women's Hospital Boston, MA United States; 5 Department of Psychiatry Harvard Medical School Boston, MA United States; 6 Center of Innovation for Complex Chronic Healthcare Edward Hines Jr. VA Hospital Hines, IL United States; 7 The Pulmonary Center Boston University School of Medicine Boston, MA United States; 8 Research and Development Service Dallas VA Medical Center Dallas, TX United States; 9 Department of Health Economics, Systems, and Policy Peter O’Donnell Jr School of Public Health The University of Texas Southwestern Medical Center Dallas, TX United States; 10 Office of Connected Care Veterans Health Administration Washington, DC United States; 11 School of Medicine University of California San Diego La Jolla, CA United States; 12 Mental Illness Research, Education, and Clinical Center Veterans Affairs Greater Los Angeles Healthcare System Los Angeles, CA United States; 13 Department of Psychiatry and Biobehavioral Sciences David Geffen School of Medicine University of California Los Angeles Los Angeles, CA United States; 14 Department of Health Law, Policy, and Management Boston University School of Public Health Boston, MA United States

**Keywords:** patient-generated health data, veterans, mobile health, mHealth, digital phenotyping, wearable electronic devices

## Abstract

**Background:**

The integration of patient-generated health data (PGHD) into health care has the potential to significantly transform patient care and clinical practice. PGHD includes health-related data created by patients, enabling the collection of health data beyond traditional health care settings. The Veterans Health Administration (VA) has taken proactive steps to incorporate PGHD into health care through its Share My Health Data (SMHD) mobile app. Launched in 2023, the SMHD app allows veterans to securely share data from their personal digital health devices with the VA for clinical and research use. However, data characterizing patients who use such tools in real-world health care systems are lacking, creating an evidence gap for implementing PGHD-informed care equitably.

**Objective:**

This study aimed to identify the characteristics of patients using the VA SMHD mobile app, which allows veterans to share PGHD with the VA.

**Methods:**

We conducted a cross-sectional analysis of veterans who began using SMHD between October 2023 and September 2024 (n=3157, “SMHD users”). We collected demographic information, including age, sex, race/ethnicity, and rurality, and clinical information, including physiological and mental health conditions, from VA administrative data. We compared characteristics of SMHD users to a 10% random sample of veterans from the same underlying administrative data cohort that had never used the app (n=632,187, “nonusers”). Statistical analyses were performed using chi-square tests, independent t tests, and multivariable regression to assess the relationship between use and key characteristics.

**Results:**

Middle-aged veterans were more likely to be SMHD users (40-49 years: odds ratio [OR] 1.55, *P*<.001; 50-59 years: OR 1.37, *P*<.001), while veterans aged 60 years and over were less likely (60-69 years: OR 0.72, *P*<.001; ≥70 years: OR 0.24, *P*<.001). Female (OR 1.23, *P*<.001) and married (OR 1.31, *P*<.001) veterans were more likely to be SMHD users than male and unmarried veterans. In contrast, Black or African American (OR 0.62, *P*<.001) and rural (OR 0.82, *P*<.001) veterans were less likely to be SMHD users than White and urban veterans. Veterans in higher-income zip codes (OR 1.36, *P*<.001) were more likely to have used the app than those in lower-income zip codes. Clinically, SMHD users were more likely to have a service-connected disability (OR 1.81, *P*<.001), multiple physiological conditions (OR 1.86, *P*<.001), and multiple mental health diagnoses (OR 1.35, *P*<.001) versus none.

**Conclusions:**

Veterans who used the SMHD app differed significantly from nonusers across several demographic and clinical characteristics. These insights identify specific demographic and clinical subgroups with higher and lower app adoption, providing an evidence base to inform targeted implementation and outreach and support strategies to promote enhanced engagement in PGHD-informed care.

## Introduction

The health care landscape is rapidly evolving with the integration of digital patient-generated health data (PGHD) into medical practice [1]. The Office of the National Coordinator for Health Information Technology defines PGHD as “health-related data created, recorded, or gathered by or from patients (or family members or other caregivers) to help address a health concern” [2]. PGHD can include health-related data, such as physical activity, vital signs, sleep, and other physiological metrics collected through wearable devices (eg, smartwatches), mobile health apps (eg, Apple Health), and other digital devices (eg, Bluetooth-enabled blood pressure cuffs).

The use of digital PGHD is part of a broader movement, sometimes called digital phenotyping [3] or personal sensing [4], to understand how to use data from personal digital devices to improve health care outcomes and personalization of treatment. Capturing health data in patients’ everyday environments has the potential to positively impact care delivery and health outcomes. These data provide frequent, objective insights into patients’ health status, offering a more complete view of health and well-being than periodic clinical encounters. This may allow for more personalized, proactive, and responsive care, especially for management of chronic conditions and preventive care [5]. In addition, PGHD has the potential to enhance patient engagement in self-management. Researchers have found many use cases for PGHD, such as predicting onset of new symptoms [6], monitoring chronic disease [7], detection of physiological and psychological conditions [8], and predicting response to treatment [9].

Despite the many use cases and opportunities offered by PGHD, health care system implementation of PGHD-informed care has been limited. Survey research suggests that many patient populations (eg, psychiatric, arthritis) have interest in digital symptom monitoring [10-12]. However, we do not yet know which patients may be most likely to actually use digital tools that support the collection of PGHD. Understanding the characteristics of these users could help app developers and clinical leaders identify what clinical populations to target for initial PGHD-informed care initiatives and also offer insight into ways to increase uptake in patient populations that show lower propensity to use these kinds of tools. Therefore, characterizing patients who adopt PGHD-sharing tools within health care systems is a critical piece of implementation science necessary for guiding rollout and tailoring support strategies.

To move toward implementing PGHD-informed care, the Veterans Health Administration (VA) launched the Share My Health Data (SMHD) mobile app in 2023 [13]. The SMHD app allows veterans to voluntarily share data from personal digital health devices, such as Bluetooth-enabled glucometers and third-party health apps, such as Apple Health, Fitbit, and Garmin [14]. Once paired with the SMHD app, veterans’ PGHD are securely stored in a dedicated VA PGHD database [15]. The contents of this database can, in turn, be applied in support of clinical care and related research. For example, the VA is currently pilot-testing the integration of these data into clinical dashboards, allowing providers to view PGHD directly from their patients during or between appointments.

VA’s SMHD app offers a unique opportunity to examine the passive digital health–monitoring initiative within a large, integrated health care system. This study compared the demographic characteristics, clinical conditions, and health care costs of SMHD users to a nationally representative random sample of veterans who have not used the app. By describing the demographic and clinical profile of SMHD users and comparing them to the broader VA population, we aimed to generate foundational evidence to guide targeted engagement strategies, identify clinical populations for pilot programs, and highlight potential disparities in digital health tool adoption. These insights can inform efforts to broaden patient participation in PGHD-informed care and support decisions about which clinical populations may be most appropriate for early implementation within the VA and in other health care systems.

## Methods

### Design

This was a retrospective, observational, cross-sectional analysis using routinely collected data from national VA data sources. This evaluation focused on SMHD users and compared characteristics of those who used the app to those who did not. The main objective was descriptive, with no intervention assignment or randomization.

### Setting

VA’s Office of Connected Care launched the SMHD app on May 1, 2023. The app was added to the VA App Store, featured on its home page, and made available to download on iOS and Android devices [16]. After downloading and accepting the end-user agreement, users can pair their digital health devices through VA’s secure authentication process. Other than instructional materials provided on the VA Mobile App Store, no additional training, outreach, or incentives were provided.

### Cohort Selection

We examined veterans who began using the app shortly after it was launched, between October 1, 2023, and September 30, 2024 (“SMHD users”) and compared them to a random sample of veterans who did not use the app prior to or during this same time period (“nonusers”).

Our analytic cohort included SMHD users, who we identified as having at least one measurement in the PGHD database submitted through the SMHD app (N=4202). This definition of “use” indicates SMHD app download, linkage to a digital health device, and initiation of data sharing; it does not measure intensity or continuity of use over time. Exclusions were nonveterans (n=62, 1.5%), those not included in the fiscal year 2023 VA Nosos risk score dataset (n=318, 7.6%) [17], or participants in early field tests of the SMHD app (n=665, 15.8%). Early field test participants were excluded to focus this evaluation on patients who discovered, downloaded, and began using the app on their own, independent of recruitment into a controlled field test.

The comparison group of nonusers was selected using a 10% random sample of veterans who were included in the fiscal year 2023 VA Nosos risk score dataset and who had never used the SMHD app prior to September 30, 2024, the end of the evaluation period. Of note, all veterans who received care in VA during fiscal year 2023 (October 1, 2022-September 30, 2023) are included in the fiscal year 2023 VA Nosos risk score dataset. This sampling approach ensured all veterans in the sample received VA or VA-paid care and allowed for sufficient statistical power to detect differences between SMHD users and nonusers, while maintaining feasibility for data processing and analysis. The final cohort consisted of 3157 SMHD users and 632,187 nonusers.

### Data Sources

We obtained SMHD app usage data from VA’s PGHD database. PGHD were extracted via secure SQL queries and validated for appropriate SMHD source linkage and timestamp accuracy. PGHD with missing timestamps or insufficient source metadata were removed prior to cohort selection. Patient demographics and clinical encounter data were obtained from the VA’s Corporate Data Warehouse (CDW). Zip code–level household income data came from the US Census Bureau American Community Survey (2023 5-year estimates) [18].

Preuse “baseline” measures of comorbidity and cost came from the fiscal year 2023 VA Nosos risk score dataset, which includes indicators for chronic physiological and mental health conditions based on the Centers for Medicare and Medicaid Services’ Hierarchical Condition Categories (HCC) [19] and a psychiatric case-mix system (PsyCMS) developed by Sloan et al [20] and later updated for *International Classification of Diseases* (ICD)-10 diagnosis codes [21]. The Nosos dataset uses diagnosis data (ICD-9 or ICD-10) from VA outpatient encounters and inpatient stays during the entire fiscal year, including VA-purchased community care data, to calculate comorbidities. Comorbidity data are combined with use data to calculate an overall “score,” which is standardized to center around 1, so a value of 1 means that the veteran is expected to have costs that are equivalent to the national average for VA patients.

### Variables

Variables were selected based on availability in the electronic medical record and prior work examining adoption and use of other digital health technologies in VA [22-24]. Demographic variables included age, race/ethnicity, sex, marital status, rurality, zip code–level household income, and service-connected disability. A service-connected disability refers to a physical or mental health condition that was incurred or aggravated during active military service and is formally recognized by VA. Clinical variables included counts of physiological and mental health conditions (eg, 0, 1, ≥2). To avoid overlap with the mental health counts, we excluded HCC codes from the overall physiological condition counts that were for mental health conditions (eg, alcohol use disorder, schizophrenia, major depressive disorder). For the mental health counts, we excluded PsyCMS codes indicating conditions in remission (eg, alcohol use remission). Relative cost outcomes were estimated using the fiscal year 2023 Nosos concurrent risk score [19].

### Data Analysis

We compared SMHD users with the random sample of nonusers across all variables of interest using means (SDs), frequencies, and percentages. Frequencies of categorical variables were compared using chi-square tests. Means of continuous variables were compared using independent *t* tests. Standardized mean differences (SMDs) were calculated for all variables to aid interpretation of the magnitude of differences between groups, with an absolute value of >0.1 conventionally considered a small but meaningful difference.

A multivariable logistic regression was used to estimate odds ratios (ORs) associated with being an SMHD user, with SEs adjusted for potential clustering of veterans within VA regions. Because our objective was descriptive and did not have an a priori hypothesis about effect moderation, we did not include any interaction terms. To avoid multicollinearity, we checked covariates’ variance inflation factor and included a term for total chronic condition counts (eg, 0, 1, ≥2) but excluded individual comorbidities in the model. As a conventional check for model fit, we reported the Hosmer-Lemeshow goodness-of-fit test (10 groups). A complete case analysis was used for the multivariable model. The proportion of missing data was small (5%), and sensitivity analyses using alternative missing data methods were not conducted.

All analyses were conducted using R version 4.4.1 (R Foundation for Statistical Computing). Key packages included *stats* for regression modeling and *dplyr* for data manipulation.

### Ethical Considerations

This work was reviewed by the Institutional Review Boards of the VA Bedford Healthcare System in Bedford, Massachusetts, and the Edward Hines Jr VA Hospital in Hines, Illinois, and designated as program evaluation for quality improvement purposes, exempting it from further oversight (VA Handbook 1058.05).

## Results

### Characteristics of SMHD Users

SMHD users differed from nonusers in several ways (Table 1). SMHD users were younger than nonusers by 9 years (users: mean 53.2, SD 13.4; nonusers: mean 62.3, SD 17.2, years), and a greater proportion were female (users: n=539, 17.1%; nonusers: n=67,401, 10.7%). In addition, race/ethnicity differed between SMHD users and nonusers. A larger proportion of SMHD users were Hispanic or Latino (users: n=322, 10.2%; nonusers: n=47,265, 7.5%), while a smaller proportion were non-Hispanic Black or African American (users: n=477, 15.1%; nonusers: n=109,459, 17.3%). Furthermore, a smaller proportion of SMHD users lived in rural areas (users: n=914, 29%; nonusers: n=214,608, 33.9%), while a larger proportion were married (users: n=1878, 59.5%; nonusers: n=349,908, 55.3%), had a service-connected disability (users: n=2791, 88.4%; nonusers: n=460,129, 72.8%), and, on average, lived in higher-income zip codes (mean household income for users: US $79,731; nonusers: US $75,071).

Table 2 presents a comparison of health conditions between SMHD users and nonusers. Clinically, SMHD users had a significantly higher burden of mental health conditions, such as posttraumatic stress disorder, depression, anxiety, adjustment disorder, sleep disorder, attention deficit disorder, and mood disorder. In contrast, they had a lower incidence of several major physiological conditions, such as chronic obstructive pulmonary disorder, heart arrhythmias, and congestive heart failure (all *P*<.001), though they showed higher rates of obesity and arthritis. Rates of alcohol dependence or abuse did not differ significantly between SMHD users and nonusers; however, rates of nicotine dependence were slightly lower among SMHD users (users: n=358, 11.3%; nonusers: n=86,180, 13.6%; *P*<.001). In terms of total VA health care costs, SMHD users had higher mean Nosos risk scores than nonusers (users: 1.13, SD 1.34; nonusers: 1.05, SD 1.64; *P*=.005), indicating VA health care costs were about 13% higher than the VA population average for SMHD users.

**Table 1:** Unadjusted differences in demographic characteristics among SMHD^a^ users and nonusers.

Characteristics		SMHD users (n=3157)	Nonusers (n=632,187)	SMD^b^		*P* value	
Age (years), mean (SD)		53.2 (13.4)	62.3 (17.2)	0.589		<.001	
**Age group (years), n (%)**					0.716		<.001
	<40	538 (17.0)	86,458 (13.7)	—^c^		—	
	40-49	765 (24.2)	72,881 (11.5)	—		—	
	50-59	852 (27.0)	90,594 (14.3)	—		—	
	60-69	561 (17.8)	117,407 (18.6)	—		—	
	>69	441 (14.0)	264,842 (41.9)	—		—	
Female sex, n (%)		539 (17.1)	67,401 (10.7)	0.186		<.001	
**Race/ethnicity, n (%)**					0.116		<.001
	White, not Hispanic	2016 (63.9)	402,055 (63.6)	—		—	
	Black or African American, not Hispanic	477 (15.1)	109,459 (17.3)	—		—	
	Hispanic	322 (10.2)	47,265 (7.5)	—		—	
	Other race, not Hispanic	107 (3.4)	19,222 (3.0)	—		—	
	Unknown	235 (7.4)	54,186 (8.6)	—		—	
Rural residence n (%)		914 (29.0)	214,608 (33.9)	0.122		<.001	
Zip code median income (US $), mean (SD)		79,731 (27,953)	75,071 (26,646)	0.171		<.001	
**Zip code median income** **(US $)** **category,** **n (%)**					0.169		<.001
	<50,000	328 (10.4)	83,355 (13.2)	—		—	
	50,000-69,999	1011 (32.0)	221,976 (35.1)	—		—	
	70,000-89,999	828 (26.6)	157,757 (25.0)	—		—	
	≥90,000	948 (30.0)	146,026 (23.1)	—		—	
Married, n (%)		1878 (59.5)	349,908 (55.3)	0.083		<.001	
Service-connected disability, n (%)		2791 (88.4)	460,129 (72.8)	0.403		<.001	

^a^SMHD: Share My Health Data.

^b^SMD: standardized mean difference.

^c^Not applicable.

**Table 2:** Unadjusted differences in health conditions among SMHD^a^ users and nonusers.

Health conditions		SMHD users (n=3157), n (%)	Nonusers (n=632,187), n (%)	SMD^b^	*P* value
Nosos Risk Score, Mean (SD)		1.13 (1.34)	1.05 (1.64)	0.055	.005
**Physiological conditions (15 most common)**					
	Diabetes with chronic complications	450 (14.3)	88,718 (14.0)	0.006	.74
	Diabetes without complication	360 (11.4)	65,279 (10.3)	0.035	.05
	Chronic obstructive pulmonary disease	185 (5.9)	63,720 (10.1)	0.156	<.001
	Specified heart arrhythmias	235 (7.4)	62,671 (9.9)	0.088	<.001
	Congestive heart failure	154 (4.9)	45,954 (7.3)	0.100	<.001
	Vascular disease	158 (5.0)	44,802 (7.1)	0.087	<.001
	Breast, prostate, and other cancers and tumors	96 (3.0)	25,993 (4.1)	0.058	.003
	Chronic kidney disease, moderate (stage 3)	94 (3.0)	24,418 (3.9)	0.049	.01
	Morbid obesity	308 (9.8)	24,068 (3.8)	0.238	<.001
	Acute renal failure	68 (2.2)	20,267 (3.2)	0.065	.001
	Rheumatoid arthritis and inflammatory connective tissue disease	149 (4.7)	20,011 (3.2)	0.080	<.001
	Cardiorespiratory failure and shock	43 (1.4)	17,325 (2.7)	0.097	<.001
	Coagulation defects and other specified hematological disorders	80 (2.5)	17,161 (2.7)	0.011	.57
	Other significant endocrine and metabolic disorders	80 (2.5)	15,280 (2.4)	0.008	.71
	Ischemic or unspecified stroke	56 (1.8)	14,715 (2.3)	0.039	.05
**Mental health conditions (15 most common)**					
	Posttraumatic stress disorder	775 (24.5)	103,665 (16.4)	0.203	<.001
	Nicotine dependence	358 (11.3)	86,180 (13.6)	0.069	<.001
	Major depressive disorder, recurrent	474 (15.0)	56,132 (8.9)	0.19	<.001
	Anxiety, not elsewhere classified	316 (10.0)	41,227 (6.5)	0.127	<.001
	Adjustment disorder	339 (10.7)	39,001 (6.2)	0.165	<.001
	Generalized anxiety	306 (9.7)	32,517 (5.1)	0.174	<.001
	Other mental health code	124 (3.9)	22,660 (3.6)	0.018	.32
	Major depressive disorder, single episode	160 (5.1)	20,080 (3.2)	0.095	<.001
	Alcohol dependence	90 (2.9)	19,126 (3.0)	0.01	.60
	Alcohol abuse	69 (2.2)	16,206 (2.6)	0.025	.20
	Dementia	8 (0.3)	14,143 (2.2)	0.18	<.001
	Sleep disorders	138 (4.4)	13,647 (2.2)	0.125	<.001
	Attention deficit disorder	216 (6.8)	13,488 (2.1)	0.229	<.001
	Mood disorder, not elsewhere classified	103 (3.3)	12,369 (2.0)	0.082	<.001
	Bipolar disorders	104 (3.3)	10,730 (1.7)	0.103	<.001
**Number of physiological conditions**				0.065	.001
	0	1500 (47.5)	306,388 (48.5)	—^c^	—
	1	831 (26.3)	149,436 (23.6)	—	—
	≥2	826 (26.2)	176,363 (27.9)	—	—
**Number of mental health conditions**				0.324	<.001
	0	1171 (37.1)	323,307 (51.1)	—	—
	1	695 (22.0)	138,703 (21.9)	—	—
	≥2	1291 (40.9)	170,177 (26.9)	—	—

^a^SMHD: Share My Health Data.

^b^SMD: standardized mean difference.

^c^Not applicable.

### Predictors of SMHD App Use

The results from the multivariable logistic regression model are presented in Table 3. Of the 635,344 patients in the cohort, 95% (n=600,590) had complete data for the multivariable model. The Hosmer-Lemeshow test indicated no evidence of lack of fit (*P*=.19), and the variance inflation factor values ranged from 1.1 to 1.5, which is significantly below the commonly used threshold of 5 to identify multicollinearity [25]. Several characteristics were significantly associated with reduced odds of being an SMHD user. Compared to younger veterans (<40 years), older veterans (>60 years) were less likely to be SMHD users (60-69 years: OR 0.72, 95% CI 0.63-0.83; ≥70 years: OR 0.24, 95% CI 0.21-0.28). Compared to White non-Hispanic veterans, Black non-Hispanic (OR 0.62, 95% CI 0.56-0.69) and other race/ethnicity (OR 0.79, 95% CI 0.65-0.97) veterans were less likely to be SMHD users. Veterans living in rural areas (OR 0.82, 95% CI 0.75-0.89) were less likely to be SMHD users compared to veterans living in more urban areas.

Conversely, several characteristics were found to be associated with increased odds of being an SMHD user. Compared to younger veterans (<40 years), middle-aged veterans were more likely to be SMHD users (40-49 years: OR 1.55, 95% CI 1.38-1.73; 50-59 years: OR 1.37, 95% CI 1.22-1.54). Female veterans (OR 1.23, 95% CI 1.11-1.35) were more likely to be SMHD users compared to male veterans, and married veterans (OR 1.31, 95% CI 1.22-1.41) were more likely to use SMHD than unmarried veterans (ie, divorced, separated, widowed, or single). In addition, compared to veterans living in zip codes with mean incomes less than US $50,000, veterans living in higher-income zip codes were more likely to be SMHD users (US $70,000-$89,999: OR 1.15, 95% CI 1.01-1.31; ≥US $90,000: OR 1.36, 95% CI 1.19-1.55). Finally, having a service-connected disability (OR 1.81, 95% CI 1.61-2.04), having one (OR 1.54, 95% CI 1.41-1.68) or two or more (OR 1.86, 95% CI 1.68-2.05) physiological conditions (vs none), and having two or more mental health conditions (OR 1.35, 95% CI 1.24-1.47) versus none were all associated with increased odds of being an SMHD user.

**Table 3:** Multivariable logistic regression model predicting odds of being an SMHD^a^ user (N=600,590).^b^

Characteristics		OR^c^ (95% CI)	*P* value
**Age group (years; reference: <40 years)**			
	40-49	1.55 (1.38-1.73)	<.001
	50-59	1.37 (1.22-1.54)	<.001
	60-69	0.72 (0.63-0.83)	<.001
	>69	0.24 (0.21-0.28)	<.001
Female (reference: male)		1.23 (1.11-1.35)	<.001
**Race/ethnicity (reference: White)**			
	Black or African American	0.62 (0.56-0.69)	<.001
	Hispanic	0.94 (0.83-1.07)	.35
	Other	0.79 (0.65-0.97)	.02
	Unknown	0.82 (0.71-0.95)	.01
Rural (reference: urban)		0.82 (0.75-0.89)	<.001
**Zip code median income category (reference: <US $50,000)**			
	50,000-69,999	1.06 (0.93-1.20)	.36
	70,000-89,999	1.15 (1.01-1.31)	.04
	≥90,000	1.36 (1.19-1.55)	<.001
Married (reference: not married)		1.31 (1.22-1.41)	<.001
Service-connected disability		1.81 (1.61-2.04)	<.001
**Number of physiological conditions (reference: 0)**			
	1	1.54 (1.41-1.68)	<.001
	≥2	1.86 (1.68-2.05)	<.001
**Number of mental health conditions (reference: 0)**			
	1	1.09 (0.99-1.20)	.09
	≥2	1.35 (1.24-1.47)	<.001

^a^SMHD: Share My Health Data.

^b^Note that 34,754 (5%) patients were excluded from the model due to missing data. SEs were adjusted for clustering at the Veterans Health Administration (VA) regional level. Hosmer-Lemeshow goodness-of-fit test: *χ*^2^_8_=11.2, *P*=.19.

^c^OR: odds ratio.

## Discussion

### Principal Findings

PGHD-guided care has the potential to be the future of medicine. Effective future implementation requires understanding which patients are open to using digital devices and other tools to collect and share PGHD. To the best of our knowledge, this is the first evaluation to look at users of a mobile app intended to support the collection and sharing of PGHD across a health care system. We found that SMHD users are more likely to be female, be married, and live in a higher-income zip code and are less likely to be aged 60 years and over, be Black or from another race, or live in a rural area. We also found that SMHD users have higher health care costs and more medical and mental health comorbidities.

Our findings about the demographic characteristics of SMHD users are similar to the literature on users of other digital health technologies. Studies on the use of other digital health technologies have also found that women and higher-income and urban-dwelling patients are more likely to use digital health technologies [26-30]. Studies have also found that patients who are from minority races (in this case non-Hispanic Black and other races), older, and divorced or separated are less likely to use digital health technologies [31-33]. These patterns may reflect broader determinants, such as differential access to technology and broadband [29,30], variations in digital health literacy, and potential questions of trust in data-sharing initiatives [31]. As PGHD tools and PGHD-informed care initiatives are designed and implemented over the coming years, it will be important to consider how to make these tools accessible to all patients to promote health and reduce health care disparities.

In addition, these findings could inform future decisions regarding what clinical populations could be appropriate targets for initial pilots of PGHD-guided care models. In this study, SMHD users were more likely to have, for example, arthritis, morbid obesity, multiple comorbidities, and mental health diagnoses. These findings corroborate those from survey studies indicating high interest in monitoring apps and sensing technologies among these populations [10-12], but until now, data on actual uptake have been limited. Thus, our findings can help inform health care systems about what patient populations to consider first, as they start to develop and test PGHD-informed care models.

Our findings indicate that SMHD users have higher health care costs and greater rates of both mental and physiological conditions compared to the general VA population. The association between app use and higher clinical complexity suggests that SMHD adopters represent a subgroup that could stand to benefit most from more precise, personalized, and proactive PGHD-informed care models. This presents a compelling opportunity to evaluate whether integrating PGHD into routine care can improve outcomes and optimize resource use within this more complex patient population.

As health care systems increasingly adopt PGHD tools, it will be important to ensure that implementation strategies account for differences in patient characteristics and technology use. Our findings highlight demographic and clinical differences between SMHD users and nonusers, which can inform targeted outreach and support efforts. Future work should explore barriers that may limit use among certain patient groups, and identify approaches to encourage broader engagement with PGHD tools across the VA population. For example, future implementation strategies for SMHD and similar tools could integrate targeted support, such as peer-led digital coaching for older adults, partnerships with rural community centers to facilitate access, and explicit messaging addressing data privacy concerns.

### Limitations

This analysis was limited to characteristics found in the VA electronic health record. For the multivariable models, we only used complete case data, which may introduce bias if data are not completely missing at random; however, the amount of missing data was relatively small (5%). Our focus was on SMHD users for sharing PGHD, and we did not evaluate the frequency, quality, or validity of the shared data.

In addition, this paper describes the use of the SMHD app before clinical displays or broader PGHD-informed care models have been systematically implemented in VA clinical settings. The connection to care received, and, consequently, the value of sharing PGHD, is likely different at this stage of implementation than it would be in the context of provider recommendation and systematic care team engagement with the PGHD shared by patients. In addition, as implementation efforts move forward, organizational factors may play a role in adoption and use and will be important to consider in future work.

It is also important to keep in mind that this was an exploratory cross-sectional analysis, and as such, it does not make any causal claims about SMHD use and subsequent health outcomes. By design, the comorbidity and cost measures represent a preuse baseline status and therefore do not capture any changes that may have occurred after the study period began. Although we used a large nationwide sample and included several relevant covariates in our model, we cannot eliminate the possibility of unobserved confounders. For example, smartphone ownership, internet access, and perceptions of data privacy and security are not available in VA administrative data. Feasibility and acceptability were inferred from use of the SMHD app; however, no direct user feedback was collected. Finally, although this evaluation draws on a large, nationwide sample of veterans, the findings may have limited generalizability to non-VA health care settings or populations.

### Conclusion

This national evaluation of the VA’s SMHD app reveals that app adopters are more likely to be middle-aged, female, married, urban-dwelling veterans with service-connected disabilities and multiple health conditions, while being less likely to be veterans who are older, Black, or from rural or lower-income areas. These findings provide the first large-scale, real-world evidence of who actively shares PGHD within an integrated health care system, moving beyond prior studies that relied primarily on patient self-report.

Understanding these adoption patterns is critical for implementation initiatives. They identify natural adopter populations for pilot programs and highlight demographic groups at risk for digital exclusion, informing the design of equitable engagement strategies. As PGHD integration evolves, ongoing monitoring of adoption trends and focused research on barriers will be essential to ensure these transformative tools benefit all patients.

Finally, organizational factors, such as local clinic promotion strategies, provider endorsement, and the degree of PGHD integration into clinical workflows, may significantly influence future adoption. Addressing these factors alongside patient-level disparities will be key to achieving scalable PGHD-informed care across VA and other health care systems.

## Abbreviations

HCC: Hierarchical Condition Categories
ICD: International Classification of Diseases
OR: odds ratio
PGHD: patient-generated health data
PsyCMS: psychiatric case-mix system
SMD: standardized mean difference
SMHD: Share My Health Data
VA: Veterans Health Administration

## Data Availability

The US Department of Veterans Affairs prohibits unauthorized sharing of data. The data used for this evaluation are not permitted to be shared outside the Veterans Health Administration (VA) firewall without a Data Use Agreement. This limitation is consistent with other studies based on VA data; however, VA data are available to researchers behind the VA firewall with an approved VA study protocol. For more information, please visit VA Information Resource Center (VIReC) [34].
